# The value of assessing suicidal ideation

**DOI:** 10.1192/bjb.2019.80

**Published:** 2020-02

**Authors:** Gethin Morgan

**Affiliations:** Emeritus Professor of Mental Health, University of Bristol. Email: hilary.howard@blueyonder.co.uk

The paper by Gibbons *et al*^1^ concerning the distress experienced by psychiatrists who lose a patient through suicide is very welcome. We all know that such an event is difficult to bear, and this paper will encourage us to get support from others if things get really difficult, rather than soldiering on alone.

In describing how hard it is in clinical practice to detect suicide risk, let alone prevent it, the authors assert that recent research based on meta-analyses provides no evidence that suicide risk assessment in clinical practice can usefully guide clinical decision-making.

I believe that such a bald overall dismissal is regrettable because it discourages acquisition and critical evaluation of relevant clinical skills. Furthermore, I submit that it is not justified in the case of psychiatric in-patient care when addressing the problem of suicide prevention in the immediate or short-term future.

Surely all would agree that in managing severe short-term risk we ignore evaluation of suicidal ideation at our peril. When less immediate short-term risk is considered, there is also much to affirm the important role of assessing suicidal ideation in guiding clinical decision-making.

We described two series of psychiatric in-patients (1982–1984, N = 27; 1991–1993, N = 18) who died by suicide either during hospital admission or within 2 months of discharge from hospital.^2^ In each of these, a high proportion of patients, 20/27 (74%) and 15/18 (83%), had discussed their suicidal ideas with members of staff in the ward during their in-patient stay. Suicidal ideation, as recorded contemporaneously in the case notes and not retrospectively, was a key clinical feature in delineating these patients, because they could not be distinguished from others in the ward across a range of behaviours. 12/27 (44%) and 9/18 (50%) showed significant clinical improvement during their in-patient stay, even though stress in the community had remained unresolved. In the 10 years that had elapsed between our two series, the proportion of patients that killed themselves after discharge from hospital increased from 7/27 (26%) to 11/18 (61%).

These findings, based as they are on two small series of suicides, must be regarded as provisional. Yet they do suggest that knowledge of suicidal ideation can be useful in guiding clinical management decisions. Clinicians should be vigilant that clinical improvement in these particular patients may be temporary and misleading, possibly related to removal from stress in the community. They should be particularly careful to ensure that such stress has been resolved, or at least contained, by planned provision of adequate ongoing support in the community if discharge from hospital is envisaged. With the increased emphasis on community care, the proportion of these patients who kill themselves after discharge from hospital is likely to have increased further since our studies took place. Our findings suggest that clinicians should be mindful of the increased hazard which is likely to be associated with premature discharge of these patients from hospital.

Our in-patient psychiatric wards should be fertile ground for the necessary further research that is needed to clarify the many other possible aetiological factors in these suicidal deaths, and so help in the development of good clinical practice. The identification and evaluation of suicidal ideation must surely have a central role in this.
